# Alpha-tocopherol and MRI Outcomes in Multiple Sclerosis – Association and Prediction

**DOI:** 10.1371/journal.pone.0054417

**Published:** 2013-01-22

**Authors:** Kristin I. Løken-Amsrud, Kjell-Morten Myhr, Søren J. Bakke, Antonie G. Beiske, Kristian S. Bjerve, Bård T. Bjørnarå, Harald Hovdal, Finn Lilleås, Rune Midgard, Tom Pedersen, Jūratė Šaltytė Benth, Øivind Torkildsen, Stig Wergeland, Trygve Holmøy

**Affiliations:** 1 Department of Neurology, Innlandet Hospital Trust, Lillehammer, Norway; 2 Institute of Clinical Medicine, University of Oslo, Oslo, Norway; 3 Norwegian Multiple Sclerosis Competence Centre, Department of Neurology, Haukeland University Hospital, Bergen, Norway; 4 Department of Clinical Medicine, University of Bergen, Bergen, Norway; 5 Kristian Gerhard Jebsen Multiple Sclerosis Research Centre, University of Bergen, Bergen, Norway; 6 Department of Neuroradiology, Oslo University Hospital, Oslo, Norway; 7 Multiple Sclerosis Centre Hakadal, Hakadal, Norway; 8 Department of Medical Biochemistry, St. Olav’s Hospital, Trondheim University Hospital, Trondheim, Norway; 9 Department of Laboratory Medicine, Children’s and Women’s Health, Norwegian University of Science and Technology, Trondheim, Norway; 10 Helsehuset Kongsberg, Kongsberg, Norway; 11 Department of Neurology, St. Olav’s Hospital, Trondheim University Hospital, Trondheim, Norway; 12 Curato Oslo, Oslo, Norway; 13 Department of Neurology, Molde Hospital, Molde, Norway; 14 Unit for Applied Clinical Research, Norwegian University of Science and Technology, Trondheim, Norway; 15 Unilabs Drammen, Drammen, Norway; 16 Helse Sør-Øst Health Services Research Centre, Akershus University Hospital, Lørenskog, Norway; 17 Department of Neurology, Akershus University Hospital, Lørenskog, Norway; University Hospital La Paz, Spain

## Abstract

**Objective:**

Alpha-tocopherol is the main vitamin E compound in humans, and has important antioxidative and immunomodulatory properties. The aim of this study was to study alpha-tocopherol concentrations and their relationship to disease activity in Norwegian multiple sclerosis (MS) patients.

**Methods:**

Prospective cohort study in 88 relapsing-remitting MS (RRMS) patients, originally included in a randomised placebo-controlled trial of omega-3 fatty acids (the OFAMS study), before and during treatment with interferon beta. The patients were followed for two years with repeated 12 magnetic resonance imaging (MRI) scans and nine serum measurements of alpha-tocopherol.

**Results:**

During interferon beta (IFNB) treatment, each 10 µmol/L increase in alpha-tocopherol reduced the odds (CI 95%) for simultaneous new T2 lesions by 36.8 (0.5–59.8) %, p = 0.048, and for combined unique activity by 35.4 (1.6–57.7) %, p = 0.042, in a hierarchical regression model. These associations were not significant prior to IFNB treatment, and were not noticeably changed by gender, age, body mass index, HLA-DRB1*15, treatment group, compliance, or the concentrations of 25-hydroxyvitamin D, retinol, neutralising antibodies against IFNB, or the omega-3 fatty acids eicosapentaenoic acid and docosahexaenoic acid. The corresponding odds for having new T1 gadolinium enhancing lesions two months later was reduced by 65.4 (16.5–85.7) %, p = 0.019, and for new T2 lesions by 61.0 (12.4–82.6) %, p = 0.023.

**Conclusion:**

During treatment with IFNB, increasing serum concentrations of alpha-tocopherol were associated with reduced odds for simultaneous and subsequent MRI disease activity in RRMS patients.

## Introduction

Vitamin E is an essential nutritional factor found in vegetable oils and margarines, vegetables, fruits, nuts and to some extent fish [1]. Natural vitamin E comprises eight different compounds, of which alpha-tocopherol is the most abundant in human blood and has the highest biological potency [2]. Vitamin E has antioxidative and immunomodulatory properties and is considered one of the most important antioxidative factors against reactive oxygen species (ROS) overload and damages from oxidative stress [3]. ROS and oxidative stress have been incriminated in the pathogenesis of several diseases, including neurodegenerative disease and multiple sclerosis (MS) [4].

Vitamin E is shown to affect different immune cells. In mice, vitamin E enhanced naïve T cell function by increasing division and interleukin-2 production, and by reducing T cell suppressive prostaglandin E_2_ from macrophages [3]. Moreover, appropriate function and interaction between CD8^+^ T cells, dendritic cells and T regulatory cells in response to viral infection depend on adequate vitamin E levels [5]. In murine microglia cultures, vitamin E has been shown to induce morphological changes and down regulate different adhesions molecules, both associated with deactivation [6]. Treatment with vitamin E also inhibited demyelination caused by ethidium bromide [7], increased subsequent remyelination (7), and has been shown to exert dose-dependent effects in a murine lupus model [8].

The effect of interferon beta (IFNB) in MS is only partial, and antioxidant therapy might potentially be an adjuvant. IFNB treatment was associated with higher concentrations of alpha-tocopherol in plasma of MS patients compared to controls [9], and also with normalization of the alpha-tocopherol levels in erythrocytes [10].

In spite of relevant biological properties, the relationship between vitamin E and disease activity in MS has not been investigated. To chart the vitamin E levels and their relationship to disease activity in Norwegian MS patients, we have measured alpha-tocopherol in serum samples from 88 relapsing-remitting MS (RRMS) patients who participated in a randomised placebo-controlled trial of supplementation with omega-3 fatty acids or placebo in RRMS patients before and during treatment with IFNB (the OFAMS study) [11]. The patients were followed for two years with repeated and corresponding magnetic resonance imaging (MRI) scans and serum sampling, as well as registration of clinical disease activity.

## Methods

The design and the results of the OFAMS study have been presented previously [11]. In brief, 88 RRMS patients were allocated to either omega-3 fatty acids (Triomar©) or placebo (corn oil), and followed for two years with repeated and corresponding MRI scans and serum sampling. None of the patients had received treatment with immunomodulatory agents six months prior to inclusion. All patients were initiated on interferon beta-1a (IFNB) at study month 6. The MRI scans were conducted monthly from baseline to study month 9, thereafter at month 12 and 24, and evaluated blindly and independently by two experienced neuro-radiologists for new T1 gadolinium (Gd^+^) enhancing lesions, new T2 lesions and combined unique activity (CUA; sum of T1Gd^+^ lesions and new or enlarging T2 lesions) as previously described [11], [12]. The serum samples were collected at baseline, thereafter at month 1, 3, 6, 7, 9, 12, 18 and 24 and analysed for alpha-tocopherol. The omega-3 fatty acids eicosapentaenoic acid (EPA) and docosahexaenoic acid (DHA) were analysed at baseline and month 6, 12 and 24. Both the omega-3 fatty acid and the placebo preparations were added vitamin E in order to protect the fatty acids against oxidation. The omega-3 fatty acid capsules contained four IU alpha-tocopherol, which equals approximately 13 mg per daily dose. The placebo preparation contained mixed tocopherols of which approximately 22 mg per daily dose were alpha-tocopherol. Neither of the preparations contained vitamin D nor A. The Expanded Disability Status Scale (EDSS) score was evaluated every sixth months and relapses were recorded throughout the study period.

### Analyses

Serum samples were stored at −80°C until analysis, which was performed simultaneously by blinded lab-technicians for all samples from each patient. The concentration of alpha-tocopherol was measured at Department of Medical Biochemistry, St. Olavs Hospital, Trondheim University Hospital, using an Agilent 100 high performance liquid chromatography (HPLC) system equipped with a diode array detector (Agilent Technologies, Santa Clara, CA USA) and the Reagent kit for the HPLC determination of alpha-tocopherol in serum (CHROMSYSTEMS Instruments & Chemicals GmbH, München, Germany). The within run coefficient of variation was 0.7% at 30.9 µmol alpha-tocopherol/L and the day-to-day coefficient of variation was 3.0% at 41.8 µmol alpha-tocopherol/L. Neutralising antibodies (NAb) against IFNB were analysed by real time quantitative polymerase chain reaction at study month 24, and categorized as negative, intermediate or high. The analyses of 25-hydoxyvitamin D, retinol, EPA, DHA and HLA-DRB1*15 has been described previously [11], [12], [13].

### Statistics

As the MRI outcomes were skewed towards none or one lesion, they were dichotomised as present or absent. We addressed our research question by using a hierarchical logistic regression model as previously described [12]. This model takes into account the repeated MRI scans and serum measurements within a patient. The SAS GLIMMIX procedure was used to fit the model with random intercepts for patients and fixed effects of alpha-tocopherol. Only paired measurements of alpha-tocopherol and MRI scans were included in the regression model. The same statistical model was used to analyse the association between alpha-tocopherol and MRI outcomes lagged by 1 and 2 study months after the alpha-tocopherol measurements. Gender, age, body mass index (BMI), HLA-DRB1*15 status, treatment group (omega-3 or placebo), compliance (intake of study medication in percentage of the total dosage) and serum concentrations of 25-hydroxyvitamin D, retinol, EPA and DHA and NAb against IFNB (categorized as negative, low to moderate and high concentration) were included as possible predictors to the logistic regression model. The Pearson’s correlation coefficient (r) was calculated to examine the association between baseline values of alpha-tocopherol and the cumulative number of new T1Gd^+^ and T2 lesions and CUA. Independent samples t-test was used for the comparison of means. Mean (SD) values are presented unless otherwise stated. The statistical analyses were conducted using SAS version 9.2 and SPSS version 15.0. Findings with p<0.05 were considered significant.

### Missing Values

Twelve alpha-tocopherol, 25-hydroxyvitamin D and retinol values were missing (one at baseline, three during study months 1–6 and eight during study months 7–24). Twenty-three MRI scans were missing (14 during study months 1–6, nine during study months 7–24). We defined MRI scans and serum samples collected within an interval of one month as paired, and 11 MRI/alpha-tocopherol measurements that exceeded this limit were excluded from analysis. EDTA-blood for HLA-DRB1 typing was missing for four, and BMI was missing for two patients. EDSS scores were missing for two patients at month 24. Missing values were not replaced.

### Ethics Statement

The study was approved by the Regional Committee for Medical and Health Research Ethics in Western Norway Regional Health Authority. All participants gave written informed consent.

## Results

### Patient Population

Eighty-eight RRMS patients, 57 (65%) women and 31 (35%) men, 58 (66%) HLA-DRB1*15 positive, age 38.9 (8.3) years, BMI 25.7 (4.3), disease duration 1.9 (3.1) years, and EDSS score at baseline of 1.9 (0.8) were included in the study.

### Alpha-tocopherol Status

**Figure 1:**
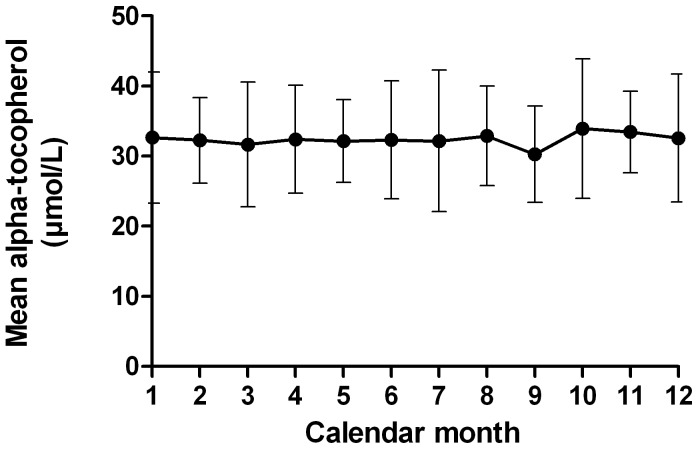
Seasonal distribution of alpha-tocopherol concentrations in serum during the whole study period. All 780 alpha-tocopherol measurements from 88 patients clustered to the calendar month of sampling. Error bars represent SD.

From the whole study period (study months 0–24) there were 780 measurements of alpha-tocopherol. The mean baseline concentration of alpha tocopherol was 29.5 (7.3) µmol/L and the mean concentration during the rest of the study was 32.4 (0.76) µmol/L (p<0.001 for difference). The mean difference between concentrations at baseline and through the rest of the study period was 2.0 (3.9) µmol/L for the patients treated with omega-3 fatty acids (n = 46) and 4.5 (3.8) µmol/L for the placebo group (n = 41) (p = 0.003 for difference). There were no significant differences before and during IFNB treatment or between genders, or any seasonal variation (Figure 1). The mean ratio between the highest and the lowest concentration of alpha-tocopherol (baseline not included) in each patient was 1.34 (0.22). The intraclass correlation coefficient was 0.788, which implies that 21.2% of the total variance in alpha-tocopherol concentrations was explained by intra-individual variation. The reference range for alpha-tocopherol is 10.5–43.5 µmol/L, and 728 (93.3%) of all the measurements were within this interval. None of the measurements were below the reference range, whereas 52 (6.7%) were above 43.5 µmol/L.

### MRI Outcomes

From the whole study period, a total of 587 paired MRI scans and alpha-tocopherol measurements (interval 3.4 (5.3) days), of which 254 were collected before and 333 after initiation of IFNB treatment, were available for analysis (table 1). During IFNB treatment, the odds (CI 95%) for new T2 lesions and CUA were significantly reduced by 36.8 (0.5–59.8) % and 35.4 (1.6–57.7) % with each 10 µmol/L increase in alpha-tocopherol, whereas no significant association was found before IFNB treatment or for the whole study period. It has previously been reported higher concentrations of alpha-tocopherol in women compared to men, and an inverse relationship with BMI [14]. Adjusting for gender, age and BMI did not noticeably change our results.

**Table 1:** Odds ratio for MRI outcomes for each 10 µmol/L increase in alpha-tocopherol.

	Whole study period (88 patients)^a^		Prior to IFNB (88 patients)^b^		During IFNB (88 patients)^c^	
MRI outcomes	Odds ratio (CI 95%)	p-value	Odds ratio (CI 95%)	p-value	Odds ratio (CI 95%)	p-value
**CUA**	0.803 (0.584–1.103)	0.176	0.732 (0.461–1.164)	0.186	0.646 (0.423–0.984)	0.042
**New T2 lesions**	0.737 (0.537–1.011)	0.059	0.682 (0.450–1.034)	0.071	0.632 (0.402–0.995)	0.048
**New T1Gd^+^ lesions**	0.838 (0.598–1.174)	0.305	0.787 (0.511–1.212)	0.275	0.651 (0.403–1.053)	0.080

a 587 alpha-tocopherol/MRI pairs,

b 254 alpha-tocopherol/MRI pairs,

c 333 alpha-tocopherol/MRI pairs.

HLA DRB1*15 is the main genetic risk factor for MS and may interact with environmental risk factors [15]. We have previously reported that vitamin D [12] and vitamin A [13] levels were inversely associated with MRI activity. Supplementation with EPA and DHA did not influence disease activity in the same patients [11], although EPA and DHA have been suggested to affect the disease course of MS in other studies [16]. Furthermore, the fat soluble vitamins and also the omega-3 fatty acid have some overlapping dietary sources [1], [17]. The associations between alpha-tocopherol and MRI outcomes were only significant during IFNB treatment. Thus, NAb against IFNB could potentially have influenced the results. As the omega-3 and placebo preparations contained alpha-tocopherol, treatment group (omega-3 or placebo) and compliance could potentially also have confounded the results. To exclude that the results were influenced by these factors, 25-hydroxyvitamin D and retinol (measured in all serum samples) and EPA and DHA (measured at month 6, 12, and 24), treatment group, compliance, and the concentration of NAb against IFNB (no, intermediate or high) were entered into the logistic regression model. Entering these variables separately or together did not significantly change the results (data not shown).

To examine whether higher alpha-tocopherol levels protect against subsequent disease activity, we also examined if the baseline concentration correlated with the cumulative number of MRI lesions during the study period. For all MRI outcomes, there was a non-significant trend for a negative correlation (CI 95%) with the baseline alpha-tocopherol level (r = −0.12 (−0.31–0.09) for new T1Gd^+^ lesions, −0.15 (−0.35–0.06) for new T2 lesions, and −0.16 (−0.36–0.05) for CUA).

To examine whether alpha-tocopherol predict subsequent MRI disease activity, we analysed the association with MRI outcomes lagged by one (31.2 (6.6) days) and two (62.1 (7.1) days) study months. During IFNB treatment, the odds (CI 95%) for new T1Gd+ lesions two months later was reduced by 65.4 (16.5–85.7) %, and for new T2 lesions by 61.0 (12.4–82.6) % with each 10 µmol/L increase in alpha-tocopherol (table 2).

**Table 2:** Odds ratio for lagged MRI outcomes for each 10 µmol/L increase in alpha-tocopherol.

		Whole study period^a^		Prior to IFNB^b^		During IFNB^c^	
MRI outcomes		Odds ratio (CI 95%)	p-value	Odds ratio (CI 95%)	p-value	Odds ratio (CI 95%)	p-value
**CUA**	**Lag 1 month**	0.835 (0.564–1.235)	0.365	0.730 (0.450–1.185)	0.202	0.878 (0.535–1.435)	0.596
	**Lag 2 months**	0.855 (0.582–1.258)	0.426	0.907 (0.562–1.463)	0.688	0.659 (0.366–1.188)	0.163
**New T2 lesions**	**Lag 1 month**	0.779 (0.541–1.122)	0.179	0.681 (0.426–1.088)	0.107	0.840 (0.508–1.390)	0.493
	**Lag 2 months**	0.773 (0.535–1.116)	0.169	0.917 (0.607–1.386)	0.680	0.390 (0.174–0.876)	0.023
**New T1Gd^+^ lesions**	**Lag 1 month**	0.738 (0.495–1.100)	0.135	0.632 (0.379–1.053)	0.078	0.824 (0.490–1.386)	0.462
	**Lag 2 months**	0.731 (0.473–1.074)	0.105	0.834 (0.525–1.325)	0.440	0.346 (0.143–0.835)	0.019

a Whole study period: Lag 1∶88 patients, 419 observations, Lag 2∶88 patients, 420 observations.

b Prior to IFNB: Lag 1∶88 patients, 250 observations, Lag 2∶88 patients, 251 observations.

c During IFNB: Lag 1∶86 patients, 169 observations, Lag 2∶87 patients, 169 observations.

### Clinical Disease Activity

The mean concentration of alpha-tocopherol was 30.95 (1.22) µmol/L in the 23 patients who experienced at least one relapse during the study period and 32.85 (0.93) µmol/L in the 65 who did not (p = 0.28). In the 26 patients who progressed at least one EDSS point the mean concentration was 31.68 (1.39) µmol/L and 32.74 (0.94) µmol/L in the 60 stable patients (p = 0.54). We did not detect any significant association between the mean concentration of alpha-tocopherol and the occurrence of relapses when stratified by IFNB treatment.

## Discussion

We found that during IFNB treatment, increasing serum concentrations of alpha-tocopherol were associated with reduced odds for simultaneous and subsequent MRI disease activity. The results were not noticeably influenced by gender, age, BMI, HLA-DRB1*15 status, treatment group or compliance (omega-3 fatty acids or placebo), or the concentrations of NAb against IFNB, 25-hydroxyvitamin D, retinol, EPA or DHA.

Studies regarding vitamin E and MS are relatively few. In small cross-sectional studies, lower concentrations of both vitamin E and vitamin E/cholesterol-ratio have been reported in stable MS patients compared to controls [18], and also in MS patients during exacerbation compared to stable MS patients with or without IFNB treatment, and controls [9], [19], [20]. In a prospective study, the risk of developing MS was not associated with total or dietary intake of vitamin E [21]. To our knowledge, there are no prospective studies addressing the relationship between vitamin E and MRI disease activity in MS.

Our patient cohort comprises well characterized RRMS patients examined with repeated MRI scans and serum measurements, allowing eight paired MRI/alpha tocopherol assessments, and is well suited for a prospective study of the relationship between vitamin E and MS disease activity. Moreover, simultaneous measurements of vitamin A, vitamin D, DHA, EPA and NAb against IFNB in the same patients, combined with records of the compliance of study medication, enabled us to adjust for potential confounding. However, our study also has limitations. Although several paired MRI scans and serum measurements of alpha-tocopherol were conducted in each patient, the cohort might have been too small to detect minor, but nevertheless potentially important associations with relapse rate and EDSS progression. The dietary habits including use of vitamin supplements were not recorded. Moreover, the patients received either approximately 13 or 22 mg alpha-tocopherol from the omega-3 or placebo preparations. This constitutes 2–3 times the intake of total vitamin E estimated in a Finnish study [22], and also the estimated average daily intake of vitamin E in Norway (8–10 alpha-tocopherol equivalents, corresponding to 8–10 mg alpha-tocopherol) [23]. Only the baseline values are therefore representative for the habitual vitamin E status of the patients. Accordingly, there was an increase in the mean concentration of alpha-tocopherol from baseline to the rest of the study period, and the supplementation might therefore have evened out both the inter- and intra-individual variation. Even so, we found a mean ratio between the highest and the lowest concentration of alpha-tocopherol in each patient of 1.34 and 22.8% of the total variation was accounted for by intra-individual variation. The serum concentration of alpha-tocopherol is correlated to the concentration of lipoproteins [2], [24]. Unfortunately, we did not measure lipoproteins or total cholesterol, and can therefore not exclude that lipoproteins might have confounded our results. However, in a previous study T2 lesion volume was not associated with lipoprotein concentrations [25], and the association with new T2 lesions was not influenced by BMI or omega-3 fatty acids in our study. It is therefore less likely that lipoprotein status have confounded the results.

Vitamin E has both antioxidative, immunomodulatory and neuroprotective properties [3], [7], [26]. Our results are therefore biologically plauisible. Immune cells produce ROS, which may contribute to neuroinflammation in experimental allergic encephalomyelitis [27] and MS [28], [29]. Vitamin E has also been shown to have other properties including regulation of enzymatic activity and gene transcription [2] that might be relevant in MS [30], [31], and to have immunomodulatory properties in animal models of rheumatoid arthritis [26] and systemic lupus erythematosus [8].

Previous small studies have reported an increase of alpha-tocopherol in erythrocytes and plasma of MS patients treated with IFNB [9], [10], as well as normalisation of ROS production in mononuclear cells [32]. The finding that the odds for new MRI disease activity were only significantly reduced during IFNB treatment could indicate an interaction between IFNB and vitamin E. However, the difference in odds reduction before and during IFNB treatment was modest, and adjusting for NAb against IFNB did not alter our results. Thus, there is not sufficient evidence to draw any conclusion, and a possible interaction between vitamin E and IFNB treatment should be studied in a larger cohort.

In conclusion, we have shown an association between increasing alpha-tocopherol concentrations and simultaneous and subsequent MRI disease activity in RRMS patients during treatment with IFNB. The relation between vitamin E and MS should be further investigated in epidemiological and experimental studies.
